# Cost‐Effectiveness Analysis of First‐Line Chemotherapy for Metastatic Pancreatic Cancer in Japan

**DOI:** 10.1002/cam4.71233

**Published:** 2025-09-15

**Authors:** Yuriko Sasahara, Yuki Takumoto, Tatsunori Murata, Manabu Akazawa, Hiroto Narimatsu

**Affiliations:** ^1^ Department of Medical Oncology Yamagata Prefectural Central Hospital Yamagata Japan; ^2^ Department of Public Health and Epidemiology Meiji Pharmaceutical University Tokyo Japan; ^3^ CRECON Medical Assessment Inc Tokyo Japan; ^4^ Cancer Prevention and Cancer Control Division Kanagawa Cancer Center Research Institute Yokohama Kanagawa Japan; ^5^ Graduate School of Health Innovation Kanagawa University of Human Services Kawasaki Kanagawa Japan; ^6^ Department of Genetic Medicine Kanagawa Cancer Center Yokohama Kanagawa Japan

**Keywords:** ICER, incremental cost‐effectiveness, QALY, S‐1, safety

## Abstract

**Backgrounds:**

Pancreatic cancer is a highly aggressive disease with limited treatment options. The combination of tegafur, gimeracil, and oteracil (S‐1) has emerged as a promising treatment approach in Japan. However, the economic implications of S‐1 compared to other chemotherapy regimens have not been fully explored.

**Methods:**

This cost‐effectiveness analysis study evaluated the economic position of S‐1 relative to fluorouracil+leucovorin+irinotecan+oxaliplatin (FFX), gemcitabine+Nab‐paclitaxel (GnP), and gemcitabine (GEM) for the treatment of distant metastatic pancreatic cancer in Japan. Incremental cost‐effectiveness ratios were calculated using quality‐adjusted life years (QALYs) as the measure of effectiveness.

**Results:**

S‐1 demonstrated the lowest incremental cost‐effectiveness ratio compared to FFX, GnP, and GEM, indicating that it offers the most value for the cost. The total cost per QALY for S‐1 was significantly lower than the other regimens. Additionally, S‐1 was associated with favourable safety and convenience profiles.

**Conclusion:**

The findings of this study suggest that S‐1 is a cost‐effective and promising treatment option for distant metastatic pancreatic cancer in Japan. Its favourable economic profile, combined with its safety and convenience advantages, makes it a viable choice for patients and healthcare providers.

## 1 Introduction

Cancer has been the leading cause of death in Japan since 1981, and pancreatic cancer is a leading cause of cancer‐related deaths in developed countries [1, 2]. The site‐specific mortality rate of pancreatic cancer is the fourth highest in Japan and increases every year [3, 4, 5]. Pancreatic cancer is difficult to detect in its early stages and is often diagnosed as unresectable owing to advanced invasion into surrounding tissues, even in the absence of distant metastases [6]. Chemotherapy is used to treat distantly metastatic pancreatic cancer. Although systemic chemotherapy is recommended by the Japanese Pancreatic Cancer Guidelines for 2022, it is difficult to cure the disease. The primary treatment goals are prolonging life and improving quality of life (QoL) [4, 7].

Primary chemotherapy for metastatic pancreatic cancer in Japan mainly includes fluorouracil+leucovorin+irinotecan+oxaliplatin (FFX), gemcitabine+Nab‐paclitaxel (GnP), tegafur+gimeracil+oteracil (S‐1), and gemcitabine (GEM). FFX and GnP are recommended as first‐line agents based on the results of clinical trials for each regimen [7, 8, 9, 10, 11, 12, 13]. In addition, modified FFX (mFFX), in which some drugs are omitted from FFX, has been used in recent years, considering the impact of side effects [7]. GEM and S‐1 monotherapies have been suggested for patients for whom other regimens are not suitable because of their general condition or age [7, 11].

In particular, S‐1 is approved only in Japan and South Korea and is the only regimen that can be administered orally [11, 12]. Therefore, the choice of regimen can vary depending on the patient's general condition, values, and wishes, and is based on the characteristics of the therapeutic agent.

A study in Japan showed that GnP had the highest proportion of prescriptions in regimens for patients with pancreatic cancer, followed by GEM, S‐1, and FFX/mFFX [14]. In 2018, the median cost of anticancer drugs per month was the highest for GnP at JPY 75,006 (USD 513) and the lowest for S‐1 at JPY 28,060 (USD 192). Therefore, S‐1 and GEM are sometimes used because of their superior safety and economic efficiency.

Although anti‐malignant tumor drugs have contributed considerably to prolonging survival by promoting their development, drug costs have skyrocketed [15, 16]. In response, the high‐cost medical care reimbursement system in Japan established a payment ceiling for medical expenses, thus reducing the burden of drug medical costs on patients [17]. In contrast, a study assessing financial toxicity in Japan using the Comprehensive Score for Financial Toxicity tool reported that several patients experienced financial toxicity during cancer treatment [18]. In addition, a cost‐effectiveness evaluation system was introduced in 2019 to assess the efficiency of medicines from a health economic evaluation perspective [19]. When conducting cost‐effectiveness analysis, drug prices are adjusted based on the evaluation results; however, there is limited evidence unique to Japan, including QoL values [20].

There have been several national and international reports on cost‐effectiveness analysis of primary chemotherapy for pancreatic cancer [21, 22, 23]. The cost‐effectiveness analysis of primary chemotherapy for metastatic pancreatic cancer by Gharaibeh et al. included GEM, FFX, GnP, GEM+oxaliplatin therapy (GEM+OX), GEM+cisplatin therapy (GEM+CIS), and GEM+capecitabine (GEM+CAP); for a total of six regimens, the incremental cost‐effectiveness ratio (ICER) was estimated with quality‐adjusted life years (QALYs) as the measure of effectiveness [21]. The results showed that GEM+CAP against GEM had the lowest ICER of £28,066/QALY, followed by FFX at £33,020/QALY. In conclusion, when the Cost/QALY threshold was set at £30,000/QALY, GEM+CAP was the most cost‐effective regimen. A Japanese cost‐effectiveness analysis comparing four regimens (FFX, GnP, GEM, and S‐1) rated GEM and S‐1 as the most cost‐effective regimens; however, they were less effective [22].

However, the study used data from 44 patients from a single center, raising concerns about the uncertainty of the results. Moriwaki et al. compared GEM and GnP and found that GnP was not cost‐effective at US$ 192,992/QALY when the threshold willingness to pay was set at US$ 68,000/QALY [23]. Nevertheless, this report calculated healthcare costs from the Japan Medical Data Center (JMDC), which did not include data for people aged 75 years and older. Additionally, the QoL values included foreign populations [24]. As noted earlier, although cost‐effectiveness analyses comparing multiple treatment regimens have been conducted in other countries, no studies have comprehensively compared the treatment regimens recommended in Japanese clinical practice guidelines. Additionally, due to differences in social conditions and healthcare systems, it is difficult to extrapolate the results from other countries to Japan, and domestic studies have raised concerns about the uncertainty of cost and QoL data.

We conducted studies on the efficacy, healthcare costs, and QoL values of chemotherapy to investigate the cost‐effectiveness in metastatic pancreatic cancer [14, 25, 26, 27]. Based on these results, the present study performed a cost‐effectiveness analysis of the treatment options recommended in the Japanese pancreatic cancer practice guideline 2022 as primary chemotherapy for metastatic pancreatic cancer to determine the economic position of each regimen. As national healthcare expenditure in Japan continues to increase and cost‐effectiveness analysis is expected to become increasingly important in approving expensive anticancer drugs, cost‐effectiveness analysis using data calculated in Japan will be valuable in the economic evaluation of regimens for pancreatic cancer in the future. The results of this study provide an opportunity to consider treatment choices from an economic perspective.

## 2 Methods

This study followed the International Society for Pharmacoeconomics and Outcomes Research's (ISPOR) good reporting practice guidelines for health economic evaluations (CHEERS checklist) [28]. The ISPOR is an international organization that promotes awareness and dissemination of pharmacoeconomics and outcome research necessary to efficiently deliver quality healthcare technologies.

### 2.1 Patient Population

**FIGURE 1:**
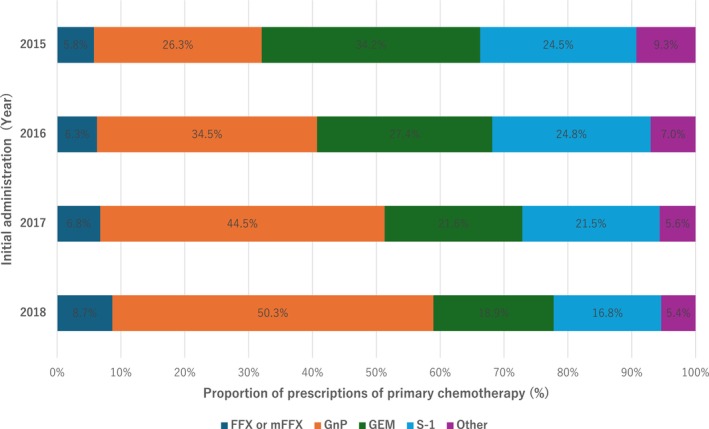
Annual trends in the prescription of first‐line chemotherapy for pancreatic cancer in Japan.

The target population for this study comprised patients receiving systemic primary chemotherapy for metastatic pancreatic cancer in Japan. The characteristics (e.g., sex and age) of the target patients included in phase III clinical trials investigating the efficacy and cost of regimens were retrieved from previous studies [8, 10, 11, 29]. Although phase III clinical trials, except for those on S‐1, were conducted outside Japan, the consistency of efficacy was ensured by single‐arm studies in Japanese subjects [9, 13]. The information on patient background obtained from the reference literature used to research the parameters of efficacy and safety, including QoL values and cost for each regimen, is summarized in Table S1. Annual trends in the prescriptions of first‐line chemotherapy for pancreatic cancer in Japan are shown in Figure 1.

### 2.2 Model Structure and Analysis Design

**FIGURE 2:**
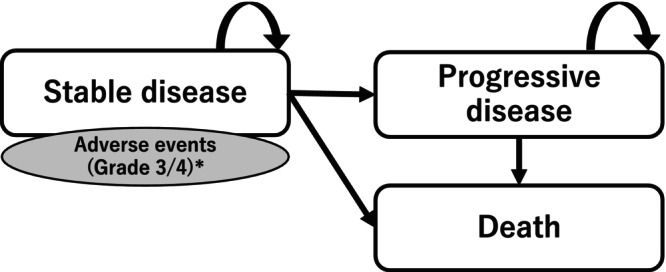
Partitioned survival model of three health states.

A partitioned survival model was developed consisting of three disease states (stable disease: SD, Progressive disease: PD, and death) (Figure 2) [30]. SD is a state in which pancreatic cancer is stable with chemotherapy, PD is a state in which the pancreatic cancer has worsened due to tumor size expansion, and no side effects are assumed because active treatment is discontinued, and death includes natural death other than death due to pancreatic cancer. The partitioned survival model is a commonly used model structure in oncological cost‐effectiveness analysis, especially by the National Institute for Health and Care Excellence (NICE) Decision Support Unit, which supports cost‐effectiveness analysis for the UK's health economic assessment organization and calculates the proportion of patients in each disease state over time. Many patients were in the SD state at the beginning of the analysis period; however, the proportion of PD and death increased over time.

The analysis cycle of this study was monthly. The analysis period was set from the start of primary chemotherapy treatment to the end of the patient's lifetime, and the starting age of the analysis model was 65 years based on the age range of patients with pancreatic cancer and the patient background in the clinical trials. The position of the analysis was that of the healthcare payer, and the discount rate was set at 2% for both costs and QALYs. These settings are based on the cost‐effectiveness evaluation guidelines followed in Japan [31].

### 2.3 Efficacy

The proportion of patients per analysis cycle was estimated from a previous network meta‐analysis (NMA) of primary chemotherapy in previous studies [25].

Although the best‐fitting functional model in the NMA was a log‐logistic distribution, the effect of the treatment intervention on SD was expected to have a considerable impact on the outcomes. Therefore, the analysis in this study was performed with a log‐normal distribution, which was slightly different from the Akaike information criterion (AIC) and Bayesian information criterion (BIC), for progression‐free survival (PFS) curves plotted using Kaplan–Meier analysis. The efficacies of FFX, GnP, and S‐1 were estimated using the hazard ratios (HRs) for overall survival (OS) and PFS in the GEM group obtained from the NMA. The probabilities of transition per month per regimen are presented in Table S2. The natural mortality rate was age‐specific in Japan in 2018 [32].

### 2.4 QoL Score

The QoL values for SD were extrapolated from a previous study, the Vignette‐based QoL research in the Japanese general population, where mean QoL score (SD) = 0.634 (0.024) and the QoL values for PD by disease scenario extrapolated from a survey was mean QoL score (SD) = 0.147 (0.521) [26, 27]. The reasons for extrapolating QoL values from different subjects were (1) cost‐effectiveness analysis allows the extrapolation of results from surveys of the general population when it is difficult to survey QoL in patients [28]; (2) although healthcare professionals tend to underestimate the impact of adverse effects on QoL values compared to patients, it is not clear whether this is because healthcare professionals tend to underestimate the impact of adverse effects on QoL values [33, 34]; and (3) because the QoL values of PD patients assessed by laypersons are considerably lower than those in previous studies on other carcinomas, it is difficult to extrapolate the results for each QoL value [35, 36]. Moreover, its interpretation is difficult [37].

The adverse effects considered for disutility were Grade 3/4 neutropenia, febrile neutropenia, diarrhoea, nausea, vomiting, and peripheral neuropathy, based on the characteristics of systemic chemotherapy for pancreatic cancer described in previous studies [26]. The frequency of adverse events was calculated from phase III clinical trials for each regimen. Additionally, the duration of adverse events was established based on the opinions of physicians and pharmacists working in specialist cancer hospitals (Table 1) [10, 11, 37].

**TABLE 1:** Input disutility parameters based on adverse events.

Adverse event	Grade	Adverse event rate				Duration (years)	Disutility (/year)
		FFX	GnP	GEM	S‐1		
Neutropenia	3/4	77.8%	70.6%	41.0%	8.8%	7	0.093
Febrile neutropenia	3/4	22.2%	5.9%	0.4%	0.4%	14	0.311
Diarrhoea	3/4	8.3%	5.9%	1.1%	5.5%	3	0.328
Nausea/vomiting	3/4	8.3%	—	0.7%	1.5%	3	0.392
Neuropathy	3/4	5.6%	11.8%	—	—	14	0.264
References		[8]	[10]	[11]	[11]		

### 2.5 Costs

The costs for each disease state were extrapolated as treatment‐related costs per regimen, with monthly direct medical costs from the start date of primary chemotherapy onwards (Table 2) [14]. Direct medical costs, such as hospitalization, administration, drugs, procedures, and laboratory costs were considered. The monthly costs per regimen were extrapolated from insurance claims data‐derived cost estimates at Diagnostic Group Classification Comprehensive Evaluation (DPC) hospitals in Japan for up to 6 months for SD and for up to 3 months for PD. This period was set because of the small number of patients who remained on the same treatment regimen over time and the high uncertainty in the cost parameter; costs after Month 7 for SD and after Month 4 for PD were assumed to be constant at 6 months for SD and 3 months for PD, respectively. Additional costs owing to death were not considered.

**TABLE 2:** Input cost parameters (USD).

Disease status	Cycle (month)	Monthly medical cost (USD^a^)							
		FFX		GnP		GEM		S‐1	
		Mean	SD	Mean	SD	Mean	SD	Mean	SD
Stable disease	1	5198	2139	5531	2074	2924	2192	1738	1762
	2	2741	1538	3129	1690	1540	1772	1119	1638
	3	2835	1860	3156	1823	1458	1490	1060	1372
	4	2459	1925	2744	1559	1409	1813	1031	1826
	5	2314	1618	2585	1449	1211	1351	917	1062
	6—	2488	1634	2742	1676	1236	1192	1106	1863
Progressive disease	1	4877	3583	4984	3468	4090	3258	2068	2452
	2	3410	3468	3622	3368	3703	3768	2231	2923
	3—	2833	3972	3132	4517	3209	3633	2080	3436

^a^ On 10 July 2025, the exchange rate of 1 USD to yen was 146.26.

Regarding the impact of side effects, as the costs reflect the actual treatment of each regimen in practice, the additional costs due to side effects associated with the regimen were judged to have been considered. Therefore, it was deemed unnecessary to consider the additional cost parameters associated with the occurrence of side effects. The exchange rate was set at 1 USD = 146.26 JPY as of 10 July 2025.

### 2.6 Statistical Analysis

In this study, the positioning of the four regimens was evaluated using an efficient frontier approach [38]. On a plane with cost on the vertical axis and effectiveness on the horizontal axis, the most efficient regimen from the origin when a straight line was drawn from the origin to a regimen was selected and judged to be the most cost‐effective regimen. The point with the lowest slope in the regimen located in the first quadrant of the point in the regimen with the smallest CER was selected. This regimen was the second most cost‐effective. This was repeated to compare the more effective regimens with the less effective regimens among the regimens with connected points.

The primary endpoint was the Cost/QALY. The secondary endpoints were the total cost of each regimen, QALY, life year, and Cost/Life year. This analysis used Microsoft Excel (Microsoft Corporation, Redmond, WA, USA) and R software, version 3.6.3 (The R Foundation for Statistical Computing, Vienna, Austria).

To conduct sensitivity analysis, the two most cost‐effective regimens were compared, and Cost/QALY was calculated by varying each parameter extrapolated from the cost‐effectiveness analysis by ±10%. In addition, scenario analyses of “PD‐QoL value as survey results for the general population (PD‐QoL value = −0.119),” “analysis with variable frequency of side effects (10‐fold),” “analysis with variable analysis period (12 months),” and “analysis with variable discount rate (3%)” were also carried out in conjunction with the results presented as a tornado diagram.

## 3 Results

### 3.1 Base‐Case Analysis

**FIGURE 3:**
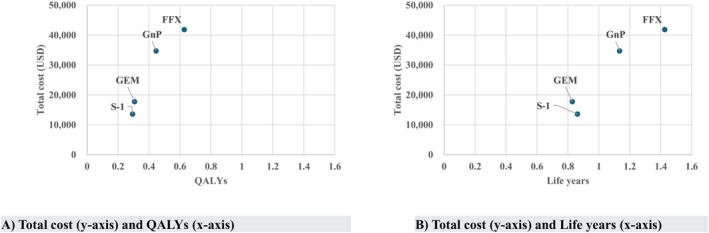
Total cost‐effectiveness plot for each regimen. (A) Total cost (*y*‐axis) and QALYs (*x*‐axis). (B) Total cost (*y*‐axis) and life years (*x*‐axis).

The analyses of the Cost/QALY, total cost, QALYs, life years, and Cost/Life years are presented in Table 3 and Figure 3. The regimen with the highest total cost was FFX, followed by GnP and GEM. The least expensive regimen was S‐1.

**TABLE 3:** Total cost, QALY, Cost/QALY, and Cost/Life year for each regimen.

Regimen	Total cost (USD^a^)	Incremental cost	QALY	Incremental QALY	Cost/QALY (USD)	Life year	Incremental life year	Cost/Life year (USD)
S‐1	13,606	—	0.294	—	—	0.862	—	—
GEM	17,751	4146	0.307	0.013	329,598	0.829	−0.033	**Dominated**
GnP	34,711	21,106	0.446	0.152	138,934	1.134	0.272	77,702
FFX	41,838	28,233	0.628	0.334	84,451	1.426	0.564	50,093

^a^ On 10 July 2025, the exchange rate of 1 USD to yen was 146.26.

The regimen with the highest QALY was FFX. It was followed by GnP and GEM, while the lowest was S‐1 (0.294). In contrast, the regimen with the lowest Cost/QALY slope was S‐1 (Figure 3A). When the cost‐effectiveness of each regimen for S‐1 was evaluated in terms of the Cost/QALY, the regimen with the lowest ratio was FFX. This was followed by GnP and GEM.

The regimen with the longest life year was FFX, followed by GnP and S‐1, whereas the regimen with the shortest life year was GEM. Similar to Cost/QALY, the regimen with the highest Cost/Life year and the lowest slope was S‐1 (Figure 3B). When the CE of each regimen for S‐1 was evaluated in terms of Cost/Life year, the regimen with the lowest ratio was FFX, followed by GnP. GEM was surpassed by S‐1, owing to GEM's increased costs and reduced effectiveness.

In addition, the comparison between regimens outside S‐1 showed that the Cost/QALY of FFX for GnP and GEM was JPY 5,714,913/QALY (USD 39,074/QALY) and JPY 10,950,155/QALY (USD 74,868/QALY), respectively (Figure S1). The Cost/Life year of FFX for GnP and GEM was JPY 3,570,038/Life year (USD 24,409/Life year) and JPY 5,904,360/Life year (USD 40,369/Life year), respectively, with similar large and small relationships.

### 3.2 Sensitivity Analysis

**FIGURE 4:**
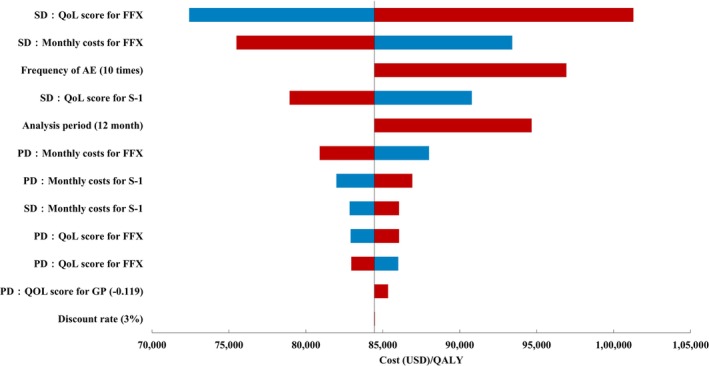
Tornado diagram for S‐1 versus FFX.

The one‐way sensitivity and scenario analysis results are presented in Figure 4 and Table S3, respectively. The Cost/QALY of FFX for S‐1, which was the most cost‐effective in terms of the efficiency frontier, was calculated; moreover, the factor with the greatest impact on the Cost/QALY was the SD QoL value in the FFX group. However, no single parameter variation could reduce the Cost/QALY of FFX for S‐1 below JPY 7.5 million/QALY (USD 51,000/QALY).

## 4 Discussion

In this study, a cost‐effectiveness analysis of the recommended regimens for primary chemotherapy for metastatic pancreatic cancer in Japan was conducted, comparing four regimens: FFX, GnP, GEM, and S‐1; S‐1 was found to be the most cost‐effective. This is because S‐1 had the lowest slope to the origin for both ratios (Figure 3). According to Japanese guidelines, FFX is not recommended for people older than 76 years, but it is recommended for the rest [7]. GEM and S‐1 are also options for patients aged < 75 years when the standard treatment is difficult due to reduced performance status. Safety, convenience, and superior cost‐effectiveness could be advantageous in selecting appropriate treatments. Based on the results depicted in Figure 3, we positioned S‐1, which had the smallest slope, as the control arm and discussed the cost‐effectiveness of other regimens compared to that of S‐1.

Contrary to the results with S‐1, QALYs and life years were the highest for FFX (Table 3). These results suggest that the biggest factor in the decline in QoL is not the toxicity of the anticancer drugs, but the progression of cancer. Primary treatment of colorectal cancer also showed a higher QoL for patients treated with bolus and infusional FU+leucovorin (LVFU2) and 5‐FU+leucovorin+oxaliplatin (FOLFOX) and LVFU2 and folinic acid+fluorouracil+irinotecan (FOLFIRI), with better QoL in the more toxic FOLFOX and FOLFIRI groups, and similar results have been reported for other carcinomas [39, 40]. Therefore, it is essential to ensure appropriate care for side effects to sustain effective toxic treatment. For example, the use of antiemetic drugs for nausea and vomiting, oral care for mouth ulcers, and other supportive therapies have been suggested to relieve symptoms and improve the QoL during treatment.

FFX is suggested as the second most cost‐effective drug. However, the Cost/QALY for FFX relative to S‐1 was considerably higher than that of the lower threshold used in the cost‐effectiveness evaluation for items requiring Japanese consideration (Table 3). Therefore, FFX exhibits poorer cost‐effectiveness compared to S‐1. This is likely because of the high cost of FFX treatment, including its side effects. In terms of Cost/QALY, even though FFX was highly effective, the QoL values for SD and PD were small, which is thought to reduce the difference in QALYs. Poorly tolerated FFX is more likely to cause severe side effects, including febrile neutropenia, and has a greater attenuating effect on QoL values. These effects are believed to increase the Cost/QALY of FFX for S‐1. However, considering the upper threshold value of JPY 15 million/QALY (USD 103,000/QALY) in the Japanese cost‐effectiveness evaluation, the Cost/QALY of FFX for S‐1 was considered acceptable. Concerning this threshold, a study by the Japanese Breast Cancer Group, which was based on a threshold study conducted on patients with breast cancer in Japan, reported differences in willingness to pay according to breast cancer severity [41]. In other words, it was not clear whether the same threshold should be used for patients with primary breast cancer and those with metastatic or recurrent breast cancer. Therefore, depending on the severity of pancreatic cancer and the purpose of treatment, there may be differences in the cost‐effectiveness threshold, i.e., the cost acceptable to patients. These considerations suggest that cost‐effectiveness should not be assessed by a uniform criterion of Cost/QALY, but rather by setting appropriate thresholds according to the disease status and end‐stage situation.

The Cost/Life year for FFX was JPY 7,32,641/Life year (USD 50,093/Life year), which is 1.5 times longer than that for S‐1 due to the longer SD and PD periods, additional costs specific to FFX, and the cost of symptomatic treatment due to side effects. Cost/Life year does not have a clear threshold. However, considering the annual drug costs of immune checkpoint inhibitors in Japan (approximately JPY 10 million, USD 68,000), the Cost/Life year of FFX for metastatic pancreatic cancer is acceptable [9, 42]. In addition, owing to the high rate of febrile neutropenia in phase II trials of FFX in Japan, mFFX use increased if the OS remains similar to that of FFX and a reduction in febrile neutropenia is allowed [9, 43]. Although the costs of both drugs are expected to be similar, a reduction in the side effects is expected to reduce the associated costs of symptomatic treatment. The cost of central venous port construction is helpful from a long‐term perspective because it is often required after secondary and palliative treatment.

Sensitivity analysis allowed for a close examination of the impact of variations in parameters on the outcomes. No single parameter significantly improved the Cost/QALY and Cost/Life year, and it was considered unlikely that variations in parameters would impact the positioning of the compared regimens (Figure 4). In contrast, the most influential parameters were the QoL values and costs of SD in FFX; hence, the Cost/QALY of FFX may be improved by reducing the consumption of healthcare resources during the treatment period with FFX in practice and by reducing the impact of disutility caused by side effects.

Compared to S‐1, GEM and GnP were unlikely to be cost‐effective, even if the threshold was varied to a certain extent. This is because the difference in QALYs is minimal for GEM compared with S‐1, as the durations of OS and PFS, which represent therapeutic efficacies, are comparable. However, it is more expensive than S‐1 because three treatment‐related hospital visits are required per month. These two factors are thought to increase the Cost/QALY. Although GnP was considered a more balanced drug owing to its higher efficacy and fewer side effects than FFX, its monthly cost was the highest and was considered to be particularly affected by drug costs.

A previous study [23] rated GnP at JPY 17,803,164/QALY (USD 121,723/QALY). Its costs were based on JMDC data, but did not include people aged over 75 years. In addition, QoL values were derived from the EQ‐5D and included overseas patients. In contrast, the present study used data that included the data of elderly patients, derived from insurance claims data from DPC hospitals in Japan, with the difference being that the QoL values were derived from a survey of the general population in Japan. The differences in the results may have arisen because the differences in incremental costs between the groups were smaller in this study than those in previous studies. In contrast, the incremental QALYs of about 0.13 for GnP compared to GEM were similar in both studies, suggesting that the interpretation of the final positions of both drugs was similar. A previous study rated GEM and S‐1 as the most cost‐effective regimens, although they were less effective [22]. In the present study, S‐1 was the most cost‐effective regimen, while GEM was the least cost‐effective regimen. Although the data in the previous study were obtained from a limited number of patients at a single center, the differences in QALYs and costs between GEM and S‐1 were minimal in this study, and GEM was similar to S‐1 in terms of efficacy and safety, making it a promising alternative to S‐1. Our model assumptions were comparable to the real‐world evidence [44].

The incidence of pancreatic cancer significantly increases from the age of approximately 60 years. As this disease predominantly affects the elderly, early detection poses considerable challenges, and numerous patients present with poor overall health conditions. The Japanese guidelines further recommend GEM or S‐1 monotherapy for patients deemed unsuitable for receiving FOLFIRINOX or GnP as a first‐line treatment due to their general health status or advanced age. Although no age limit exists for receiving FOLFIRINOX, it is not typically recommended for patients aged 76 years and older. Because many patients diagnosed with pancreatic cancer are elderly or exhibit compromised performance status, we contend that S‐1 holds significant relevance in routine clinical practice.

In practice, treatment selection in Japan has prioritized regimen efficacy, safety, and impact on QoL, and the results of economic evaluation have not been given much consideration [7, 45]. This trend is not limited to metastatic pancreatic cancer. In a preference study involving Japanese patients with EGFR‐positive non‐small cell lung cancer and their doctors, both patients and doctors responded that PFS and overall response rate were the most important factors driving their treatment preferences, whereas annual cost ranked lower in priority than moderate gastrointestinal symptoms and skin‐related symptoms [46]. In Japan, where there is a universal health insurance system, clinical treatment options are chosen based on a combination of factors, including efficacy, safety, cost‐effectiveness, and convenience. However, in the case of serious diseases including metastatic pancreatic cancer, the priority may be heavily biased toward some of these factors. Moreover, considering that Japan faces a serious increase in healthcare costs, it is necessary to ensure the sustainability of healthcare and consider the efficient use of healthcare resources [47].

### 4.1 Limitations

Several research limitations exist in this study. First, efficacy parameters derived from clinical trials may not fully represent real‐world practice. For example, the impact of secondary treatment was not considered in this study. However, the cost‐effectiveness analysis of first‐line treatment is meaningful because it is sometimes difficult for patients with pancreatic cancer having a poor prognosis to undergo second‐line treatment. Moreover, the analysis did not include emerging treatments such as mFFX and S‐IROX (S‐1, irinotecan, oxaliplatin), which were compared with GnP in terms of superiority in the JCOG1611 trial, and NALIRIFOX, which showed superiority over GnP in the NAPOLI‐3 trial [48, 49]. Second, the model excluded biomarker‐guided therapies, which may impact overall cost‐effectiveness in clinical practice. Therapeutic methods for pancreatic cancer patients with specific genetic mutations, such as BRCA1 or BRCA2 mutations and KRAS mutations, are currently being researched [50]. As these methods are established, it will be necessary to recalculate the impact of testing costs. Third, QoL values were derived from general population surveys rather than patient‐reported outcomes, though this approach follows established guidelines for cost‐effectiveness analysis [31, 51, 52]. Few studies have collected QoL values directly from patients with metastatic pancreatic cancer in Japan, and no reports of QoL values for Japanese patients could be used in this study. Finally, the exchange rate used (as of July 2025) may limit the current international interpretation of USD‐converted costs due to subsequent currency fluctuations.

## 5 Conclusion

The economic position of representative Japanese primary chemotherapy regimens for metastatic pancreatic cancer has been clarified. In particular, S‐1, which has been considered to have advantages in terms of safety and convenience, was found to be a beneficial regimen in terms of efficiency in terms of QoL values and costs. Future cost‐effectiveness analysis reflecting changes in treatments may help validate our findings.

## Author Contributions

**Yuriko Sasahara:** conceptualization (equal), writing – original draft (lead), writing – review and editing (equal). **Yuki Takumoto:** conceptualization (equal), formal analysis (equal), investigation (equal), resources (equal), software (equal), validation (equal), writing – original draft (lead), writing – review and editing (equal). **Tatsunori Murata:** conceptualization (equal), writing – original draft (equal), writing – review and editing (equal). **Manabu Akazawa:** conceptualization (equal), funding acquisition (lead), resources (equal), supervision (equal), writing – original draft (equal), writing – review and editing (equal). **Hiroto Narimatsu:** conceptualization (equal), supervision (equal), writing – original draft (equal), writing – review and editing (equal).

## Ethics Statement

The authors have nothing to report.

## Consent

The authors have nothing to report.

## Conflicts of Interest

The authors declare no conflicts of interest.

## Supporting information


**Data S1:** cam471233‐sup‐0001‐Supinfo.docx.

## Data Availability

Our data are available only to the Department of Public Health and Epidemiology, Meiji Pharmaceutical University.
