# Supplementary material for: Cost‐Effectiveness Analysis of First‐Line Chemotherapy for Metastatic Pancreatic Cancer in Japan
Source: Cancer Med. 2025 Sep 15;14(18):e71233. doi: 10.1002/cam4.71233 (PMC12434479; doi:10.1002/cam4.71233)
Supplement: Supplementary file 1 — Data S1: cam471233‐sup‐0001‐Supinfo.docx. [file CAM4-14-e71233-s001.docx]

| **Supplemental document: Table S1. Population criteria summary of each parameter for the CEA model** | | | |  |
| --- | --- | --- | --- | --- |
| Parameter categories | FFX | GnP | GEM | S-1 |
| Efficacy  (OS and PFS) | Metastatic pancreatic cancer patients with an ECOG performance status score of 0 or 1 to receive FOLFIRINOX [8] | Metastatic pancreatic cancer patients with a Karnofsky performance status score of 70 or more to receive GnP [10] | Metastatic and locally advanced pancreatic cancer patients receiving GEM [25] | Metastatic and locally advanced pancreatic cancer patients receiving S-1 [11] |
| Safety including QoL score | Metastatic pancreatic cancer patients with an ECOG performance status score of 0 or 1 to receive FOLFIRINOX [8] | Metastatic pancreatic cancer patients with a Karnofsky performance status score of 70 or more to receive GnP [10] | Metastatic and locally advanced pancreatic cancer patients receiving GEM [11] | Metastatic and locally advanced pancreatic cancer patients receiving S-1 [11] |
| Cost | Pancreatic cancer diagnosis and received at least one systemic chemotherapy including FOLFIRINOX, GnP, GEM, and S-1. No patients who underwent surgery or radiotherapy after the date of first confirmed diagnosis of pancreatic cancer [14] | | | |

| **Supplemental document: Table S2. Monthly proportion of patients undergoing chemotherapy by treatment regimen** | | | | | | | | |
| --- | --- | --- | --- | --- | --- | --- | --- | --- |
| **Cycle (month)** | **OS** | | | | **PFS** | | | |
|  | **FFX** | **GnP** | **GEM** | **S-1** | **FFX** | **GnP** | **GEM** | **S-1** |
| 1 | 99.03% | 97.14% | 93.03% | 91.63% | 99.76% | 99.45% | 98.48% | 98.65% |
| 2 | 93.91% | 86.71% | 75.45% | 72.28% | 97.80% | 95.91% | 91.35% | 92.08% |
| 3 | 86.11% | 74.24% | 58.98% | 55.15% | 93.90% | 89.83% | 81.43% | 82.68% |
| 4 | 77.57% | 62.67% | 45.99% | 42.14% | 88.75% | 82.63% | 71.27% | 72.87% |
| 5 | 69.27% | 52.75% | 36.13% | 32.55% | 83.04% | 75.22% | 61.92% | 63.72% |
| 6 | 61.64% | 44.49% | 28.69% | 25.46% | 77.18% | 68.11% | 53.69% | 55.57% |
| 7 | 54.79% | 37.67% | 23.02% | 20.17% | 71.45% | 61.51% | 46.59% | 48.48% |
| 8 | 48.74% | 32.05% | 18.67% | 16.16% | 65.99% | 55.50% | 40.52% | 42.37% |
| 9 | 43.41% | 27.41% | 15.28% | 13.09% | 60.87% | 50.08% | 35.34% | 37.12% |
| 10 | 38.75% | 23.56% | 12.62% | 10.71% | 56.13% | 45.23% | 30.92% | 32.62% |
| 11 | 34.66% | 20.36% | 10.51% | 8.84% | 51.76% | 40.91% | 27.15% | 28.75% |
| 12 | 31.08% | 17.67% | 8.82% | 7.36% | 47.75% | 37.05% | 23.92% | 25.42% |
| 13 | 27.94% | 15.41% | 7.45% | 6.17% | 44.08% | 33.61% | 21.15% | 22.55% |
| 14 | 25.17% | 13.49% | 6.33% | 5.21% | 40.72% | 30.55% | 18.75% | 20.06% |
| 15 | 22.73% | 11.86% | 5.41% | 4.42% | 37.66% | 27.81% | 16.68% | 17.90% |
| 16 | 20.58% | 10.46% | 4.65% | 3.78% | 34.86% | 25.36% | 14.89% | 16.02% |
| 17 | 18.67% | 9.26% | 4.02% | 3.24% | 32.30% | 23.17% | 13.32% | 14.37% |
| 18 | 16.97% | 8.23% | 3.49% | 2.80% | 29.97% | 21.21% | 11.95% | 12.93% |
| 19 | 15.46% | 7.33% | 3.04% | 2.43% | 27.83% | 19.44% | 10.75% | 11.65% |
| 20 | 14.11% | 6.55% | 2.66% | 2.11% | 25.88% | 17.85% | 9.69% | 10.53% |
| 21 | 12.91% | 5.87% | 2.33% | 1.84% | 24.09% | 16.42% | 8.76% | 9.54% |
| 22 | 11.82% | 5.28% | 2.06% | 1.62% | 22.44% | 15.12% | 7.93% | 8.66% |
| 23 | 10.85% | 4.76% | 1.82% | 1.42% | 20.94% | 13.95% | 7.20% | 7.88% |
| 24 | 9.98% | 4.29% | 1.61% | 1.26% | 19.55% | 12.88% | 6.55% | 7.18% |
| 25 | 9.19% | 3.89% | 1.43% | 1.11% | 18.27% | 11.92% | 5.97% | 6.56% |
| 26 | 8.47% | 3.53% | 1.28% | 0.99% | 17.10% | 11.04% | 5.45% | 6.00% |
| 27 | 7.82% | 3.20% | 1.14% | 0.88% | 16.01% | 10.24% | 4.99% | 5.49% |
| 28 | 7.23% | 2.92% | 1.03% | 0.79% | 15.01% | 9.51% | 4.57% | 5.04% |
| 29 | 6.70% | 2.66% | 0.92% | 0.71% | 14.08% | 8.84% | 4.19% | 4.64% |
| 30 | 6.21% | 2.43% | 0.83% | 0.63% | 13.22% | 8.23% | 3.85% | 4.27% |
| 31 | 5.77% | 2.23% | 0.75% | 0.57% | 12.43% | 7.67% | 3.55% | 3.93% |
| 32 | 5.36% | 2.04% | 0.68% | 0.51% | 11.69% | 7.15% | 3.27% | 3.63% |
| 33 | 4.99% | 1.88% | 0.61% | 0.46% | 11.01% | 6.68% | 3.02% | 3.36% |
| 34 | 4.65% | 1.72% | 0.56% | 0.42% | 10.37% | 6.24% | 2.79% | 3.11% |
| 35 | 4.34% | 1.59% | 0.51% | 0.38% | 9.78% | 5.84% | 2.58% | 2.88% |
| 36 | 4.05% | 1.47% | 0.46% | 0.35% | 9.23% | 5.47% | 2.39% | 2.67% |
| 37 | 3.79% | 1.35% | 0.42% | 0.31% | 8.72% | 5.13% | 2.22% | 2.48% |
| 38 | 3.54% | 1.25% | 0.39% | 0.29% | 8.24% | 4.81% | 2.06% | 2.31% |
| 39 | 3.32% | 1.16% | 0.35% | 0.26% | 7.79% | 4.52% | 1.92% | 2.15% |
| 40 | 3.11% | 1.07% | 0.32% | 0.24% | 7.38% | 4.25% | 1.78% | 2.00% |
| 41 | 2.92% | 1.00% | 0.30% | 0.22% | 6.98% | 4.00% | 1.66% | 1.87% |
| 42 | 2.74% | 0.93% | 0.27% | 0.20% | 6.62% | 3.76% | 1.55% | 1.75% |
| 43 | 2.58% | 0.86% | 0.25% | 0.18% | 6.28% | 3.55% | 1.45% | 1.63% |
| 44 | 2.42% | 0.80% | 0.23% | 0.17% | 5.95% | 3.34% | 1.35% | 1.53% |
| 45 | 2.28% | 0.75% | 0.21% | 0.16% | 5.65% | 3.15% | 1.26% | 1.43% |
| 46 | 2.15% | 0.70% | 0.20% | 0.14% | 5.37% | 2.98% | 1.18% | 1.34% |
| 47 | 2.03% | 0.65% | 0.18% | 0.13% | 5.10% | 2.81% | 1.11% | 1.26% |
| 48 | 1.91% | 0.61% | 0.17% | 0.12% | 4.85% | 2.66% | 1.04% | 1.18% |
| 49 | 1.80% | 0.57% | 0.16% | 0.11% | 4.62% | 2.52% | 0.98% | 1.11% |
| 50 | 1.70% | 0.53% | 0.15% | 0.11% | 4.40% | 2.38% | 0.92% | 1.04% |
| 51 | 1.61% | 0.50% | 0.14% | 0.10% | 4.19% | 2.26% | 0.86% | 0.98% |
| 52 | 1.52% | 0.47% | 0.13% | 0.09% | 3.99% | 2.14% | 0.81% | 0.92% |
| 53 | 1.44% | 0.44% | 0.12% | 0.08% | 3.81% | 2.03% | 0.76% | 0.87% |
| 54 | 1.37% | 0.41% | 0.11% | 0.08% | 3.63% | 1.93% | 0.72% | 0.82% |
| 55 | 1.30% | 0.39% | 0.10% | 0.07% | 3.46% | 1.83% | 0.68% | 0.77% |
| 56 | 1.23% | 0.37% | 0.09% | 0.07% | 3.31% | 1.74% | 0.64% | 0.73% |
| 57 | 1.17% | 0.34% | 0.09% | 0.06% | 3.16% | 1.65% | 0.60% | 0.69% |
| 58 | 1.11% | 0.32% | 0.08% | 0.06% | 3.02% | 1.57% | 0.57% | 0.65% |
| 59 | 1.05% | 0.31% | 0.08% | 0.06% | 2.89% | 1.49% | 0.54% | 0.62% |
| 60 | 1.00% | 0.29% | 0.07% | 0.05% | 2.76% | 1.42% | 0.51% | 0.58% |
| 61 | 0.95% | 0.27% | 0.07% | 0.05% | 2.64% | 1.35% | 0.48% | 0.55% |
| 62 | 0.90% | 0.26% | 0.06% | 0.05% | 2.53% | 1.29% | 0.46% | 0.52% |
| 63 | 0.86% | 0.24% | 0.06% | 0.04% | 2.42% | 1.23% | 0.43% | 0.50% |
| 64 | 0.82% | 0.23% | 0.06% | 0.04% | 2.32% | 1.17% | 0.41% | 0.47% |
| 65 | 0.78% | 0.22% | 0.05% | 0.04% | 2.22% | 1.12% | 0.39% | 0.45% |
| 66 | 0.74% | 0.21% | 0.05% | 0.03% | 2.13% | 1.07% | 0.37% | 0.42% |
| 67 | 0.71% | 0.19% | 0.05% | 0.03% | 2.04% | 1.02% | 0.35% | 0.40% |
| 68 | 0.68% | 0.18% | 0.04% | 0.03% | 1.96% | 0.97% | 0.33% | 0.38% |
| 69 | 0.65% | 0.17% | 0.04% | 0.03% | 1.88% | 0.93% | 0.32% | 0.36% |
| 70 | 0.62% | 0.17% | 0.04% | 0.03% | 1.81% | 0.89% | 0.30% | 0.35% |
| 71 | 0.59% | 0.16% | 0.04% | 0.03% | 1.74% | 0.85% | 0.29% | 0.33% |
| 72 | 0.56% | 0.15% | 0.03% | 0.02% | 1.67% | 0.82% | 0.27% | 0.31% |
| 73 | 0.54% | 0.14% | 0.03% | 0.02% | 1.60% | 0.78% | 0.26% | 0.30% |
| 74 | 0.52% | 0.14% | 0.03% | 0.02% | 1.54% | 0.75% | 0.25% | 0.29% |
| 75 | 0.49% | 0.13% | 0.03% | 0.02% | 1.48% | 0.72% | 0.23% | 0.27% |
| 76 | 0.47% | 0.12% | 0.03% | 0.02% | 1.42% | 0.69% | 0.22% | 0.26% |
| 77 | 0.45% | 0.12% | 0.03% | 0.02% | 1.37% | 0.66% | 0.21% | 0.25% |
| 78 | 0.43% | 0.11% | 0.02% | 0.02% | 1.32% | 0.63% | 0.20% | 0.24% |
| 79 | 0.41% | 0.11% | 0.02% | 0.02% | 1.27% | 0.60% | 0.19% | 0.23% |
| 80 | 0.40% | 0.10% | 0.02% | 0.02% | 1.22% | 0.58% | 0.19% | 0.22% |
| 81 | 0.38% | 0.10% | 0.02% | 0.01% | 1.18% | 0.56% | 0.18% | 0.21% |
| 82 | 0.37% | 0.09% | 0.02% | 0.01% | 1.14% | 0.54% | 0.17% | 0.20% |
| 83 | 0.35% | 0.09% | 0.02% | 0.01% | 1.09% | 0.51% | 0.16% | 0.19% |
| 84 | 0.34% | 0.08% | 0.02% | 0.01% | 1.06% | 0.49% | 0.15% | 0.18% |
| 85 | 0.32% | 0.08% | 0.02% | 0.01% | 1.02% | 0.47% | 0.15% | 0.17% |
| 86 | 0.31% | 0.08% | 0.02% | 0.01% | 0.98% | 0.46% | 0.14% | 0.17% |
| 87 | 0.30% | 0.07% | 0.02% | 0.01% | 0.95% | 0.44% | 0.14% | 0.16% |
| 88 | 0.29% | 0.07% | 0.01% | 0.01% | 0.91% | 0.42% | 0.13% | 0.15% |
| 89 | 0.28% | 0.07% | 0.01% | 0.01% | 0.88% | 0.41% | 0.12% | 0.15% |
| 90 | 0.26% | 0.06% | 0.01% | 0.01% | 0.85% | 0.39% | 0.12% | 0.14% |
| 91 | 0.25% | 0.06% | 0.01% | 0.01% | 0.82% | 0.38% | 0.11% | 0.13% |
| 92 | 0.25% | 0.06% | 0.01% | 0.01% | 0.79% | 0.36% | 0.11% | 0.13% |
| 93 | 0.24% | 0.06% | 0.01% | 0.01% | 0.77% | 0.35% | 0.10% | 0.12% |
| 94 | 0.23% | 0.05% | 0.01% | 0.01% | 0.74% | 0.34% | 0.10% | 0.12% |
| 95 | 0.22% | 0.05% | 0.01% | 0.01% | 0.72% | 0.32% | 0.10% | 0.11% |
| 96 | 0.21% | 0.05% | 0.01% | 0.01% | 0.69% | 0.31% | 0.09% | 0.11% |
| 97 | 0.20% | 0.05% | 0.01% | 0.01% | 0.67% | 0.30% | 0.09% | 0.10% |
| 98 | 0.20% | 0.05% | 0.01% | 0.01% | 0.65% | 0.29% | 0.09% | 0.10% |
| 99 | 0.19% | 0.04% | 0.01% | 0.01% | 0.63% | 0.28% | 0.08% | 0.10% |
| 100 | 0.18% | 0.04% | 0.01% | 0.01% | 0.61% | 0.27% | 0.08% | 0.09% |
| 101 | 0.18% | 0.04% | 0.01% | 0.01% | 0.59% | 0.26% | 0.08% | 0.09% |
| 102 | 0.17% | 0.04% | 0.01% | 0.01% | 0.57% | 0.25% | 0.07% | 0.09% |
| 103 | 0.16% | 0.04% | 0.01% | 0.00% | 0.55% | 0.24% | 0.07% | 0.08% |
| 104 | 0.16% | 0.04% | 0.01% | 0.00% | 0.53% | 0.23% | 0.07% | 0.08% |
| 105 | 0.15% | 0.03% | 0.01% | 0.00% | 0.52% | 0.23% | 0.06% | 0.08% |
| 106 | 0.15% | 0.03% | 0.01% | 0.00% | 0.50% | 0.22% | 0.06% | 0.07% |
| 107 | 0.14% | 0.03% | 0.01% | 0.00% | 0.49% | 0.21% | 0.06% | 0.07% |
| 108 | 0.14% | 0.03% | 0.01% | 0.00% | 0.47% | 0.20% | 0.06% | 0.07% |
| 109 | 0.13% | 0.03% | 0.01% | 0.00% | 0.46% | 0.20% | 0.06% | 0.07% |
| 110 | 0.13% | 0.03% | 0.01% | 0.00% | 0.44% | 0.19% | 0.05% | 0.06% |
| 111 | 0.12% | 0.03% | 0.01% | 0.00% | 0.43% | 0.19% | 0.05% | 0.06% |
| 112 | 0.12% | 0.03% | 0.00% | 0.00% | 0.42% | 0.18% | 0.05% | 0.06% |
| 113 | 0.12% | 0.02% | 0.00% | 0.00% | 0.40% | 0.17% | 0.05% | 0.06% |
| 114 | 0.11% | 0.02% | 0.00% | 0.00% | 0.39% | 0.17% | 0.05% | 0.05% |
| 115 | 0.11% | 0.02% | 0.00% | 0.00% | 0.38% | 0.16% | 0.04% | 0.05% |
| 116 | 0.10% | 0.02% | 0.00% | 0.00% | 0.37% | 0.16% | 0.04% | 0.05% |
| 117 | 0.10% | 0.02% | 0.00% | 0.00% | 0.36% | 0.15% | 0.04% | 0.05% |
| 118 | 0.10% | 0.02% | 0.00% | 0.00% | 0.35% | 0.15% | 0.04% | 0.05% |
| 119 | 0.09% | 0.02% | 0.00% | 0.00% | 0.34% | 0.14% | 0.04% | 0.05% |
| 120 | 0.09% | 0.02% | 0.00% | 0.00% | 0.33% | 0.14% | 0.04% | 0.04% |
| 121 | 0.09% | 0.02% | 0.00% | 0.00% | 0.32% | 0.13% | 0.04% | 0.04% |
| 122 | 0.09% | 0.02% | 0.00% | 0.00% | 0.31% | 0.13% | 0.03% | 0.04% |
| 123 | 0.08% | 0.02% | 0.00% | 0.00% | 0.30% | 0.13% | 0.03% | 0.04% |
| 124 | 0.08% | 0.02% | 0.00% | 0.00% | 0.29% | 0.12% | 0.03% | 0.04% |
| 125 | 0.08% | 0.02% | 0.00% | 0.00% | 0.28% | 0.12% | 0.03% | 0.04% |
| 126 | 0.08% | 0.02% | 0.00% | 0.00% | 0.28% | 0.11% | 0.03% | 0.04% |
| 127 | 0.07% | 0.02% | 0.00% | 0.00% | 0.27% | 0.11% | 0.03% | 0.04% |
| 128 | 0.07% | 0.01% | 0.00% | 0.00% | 0.26% | 0.11% | 0.03% | 0.03% |
| 129 | 0.07% | 0.01% | 0.00% | 0.00% | 0.25% | 0.10% | 0.03% | 0.03% |
| 130 | 0.07% | 0.01% | 0.00% | 0.00% | 0.25% | 0.10% | 0.03% | 0.03% |
| 131 | 0.07% | 0.01% | 0.00% | 0.00% | 0.24% | 0.10% | 0.03% | 0.03% |
| 132 | 0.06% | 0.01% | 0.00% | 0.00% | 0.23% | 0.10% | 0.02% | 0.03% |
| 133 | 0.06% | 0.01% | 0.00% | 0.00% | 0.23% | 0.09% | 0.02% | 0.03% |
| 134 | 0.06% | 0.01% | 0.00% | 0.00% | 0.22% | 0.09% | 0.02% | 0.03% |
| 135 | 0.06% | 0.01% | 0.00% | 0.00% | 0.22% | 0.09% | 0.02% | 0.03% |
| 136 | 0.06% | 0.01% | 0.00% | 0.00% | 0.21% | 0.09% | 0.02% | 0.03% |
| 137 | 0.05% | 0.01% | 0.00% | 0.00% | 0.20% | 0.08% | 0.02% | 0.03% |
| 138 | 0.05% | 0.01% | 0.00% | 0.00% | 0.20% | 0.08% | 0.02% | 0.02% |
| 139 | 0.05% | 0.01% | 0.00% | 0.00% | 0.19% | 0.08% | 0.02% | 0.02% |
| 140 | 0.05% | 0.01% | 0.00% | 0.00% | 0.19% | 0.08% | 0.02% | 0.02% |
| 141 | 0.05% | 0.01% | 0.00% | 0.00% | 0.18% | 0.07% | 0.02% | 0.02% |
| 142 | 0.05% | 0.01% | 0.00% | 0.00% | 0.18% | 0.07% | 0.02% | 0.02% |
| 143 | 0.05% | 0.01% | 0.00% | 0.00% | 0.17% | 0.07% | 0.02% | 0.02% |
| 144 | 0.04% | 0.01% | 0.00% | 0.00% | 0.17% | 0.07% | 0.02% | 0.02% |
| 145 | 0.04% | 0.01% | 0.00% | 0.00% | 0.17% | 0.07% | 0.02% | 0.02% |
| 146 | 0.04% | 0.01% | 0.00% | 0.00% | 0.16% | 0.06% | 0.02% | 0.02% |
| 147 | 0.04% | 0.01% | 0.00% | 0.00% | 0.16% | 0.06% | 0.02% | 0.02% |
| 148 | 0.04% | 0.01% | 0.00% | 0.00% | 0.15% | 0.06% | 0.02% | 0.02% |
| 149 | 0.04% | 0.01% | 0.00% | 0.00% | 0.15% | 0.06% | 0.01% | 0.02% |
| 150 | 0.04% | 0.01% | 0.00% | 0.00% | 0.15% | 0.06% | 0.01% | 0.02% |
| 151 | 0.04% | 0.01% | 0.00% | 0.00% | 0.14% | 0.06% | 0.01% | 0.02% |
| 152 | 0.04% | 0.01% | 0.00% | 0.00% | 0.14% | 0.05% | 0.01% | 0.02% |
| 153 | 0.03% | 0.01% | 0.00% | 0.00% | 0.14% | 0.05% | 0.01% | 0.02% |
| 154 | 0.03% | 0.01% | 0.00% | 0.00% | 0.13% | 0.05% | 0.01% | 0.02% |
| 155 | 0.03% | 0.01% | 0.00% | 0.00% | 0.13% | 0.05% | 0.01% | 0.01% |
| 156 | 0.03% | 0.01% | 0.00% | 0.00% | 0.13% | 0.05% | 0.01% | 0.01% |
| 157 | 0.03% | 0.01% | 0.00% | 0.00% | 0.12% | 0.05% | 0.01% | 0.01% |
| 158 | 0.03% | 0.01% | 0.00% | 0.00% | 0.12% | 0.05% | 0.01% | 0.01% |
| 159 | 0.03% | 0.01% | 0.00% | 0.00% | 0.12% | 0.05% | 0.01% | 0.01% |
| 160 | 0.03% | 0.01% | 0.00% | 0.00% | 0.11% | 0.04% | 0.01% | 0.01% |
| 161 | 0.03% | 0.01% | 0.00% | 0.00% | 0.11% | 0.04% | 0.01% | 0.01% |
| 162 | 0.03% | 0.00% | 0.00% | 0.00% | 0.11% | 0.04% | 0.01% | 0.01% |
| 163 | 0.03% | 0.00% | 0.00% | 0.00% | 0.11% | 0.04% | 0.01% | 0.01% |
| 164 | 0.03% | 0.00% | 0.00% | 0.00% | 0.10% | 0.04% | 0.01% | 0.01% |
| 165 | 0.03% | 0.00% | 0.00% | 0.00% | 0.10% | 0.04% | 0.01% | 0.01% |
| 166 | 0.02% | 0.00% | 0.00% | 0.00% | 0.10% | 0.04% | 0.01% | 0.01% |
| 167 | 0.02% | 0.00% | 0.00% | 0.00% | 0.10% | 0.04% | 0.01% | 0.01% |
| 168 | 0.02% | 0.00% | 0.00% | 0.00% | 0.09% | 0.04% | 0.01% | 0.01% |
| 169 | 0.02% | 0.00% | 0.00% | 0.00% | 0.09% | 0.04% | 0.01% | 0.01% |
| 170 | 0.02% | 0.00% | 0.00% | 0.00% | 0.09% | 0.03% | 0.01% | 0.01% |
| 171 | 0.02% | 0.00% | 0.00% | 0.00% | 0.09% | 0.03% | 0.01% | 0.01% |
| 172 | 0.02% | 0.00% | 0.00% | 0.00% | 0.09% | 0.03% | 0.01% | 0.01% |
| 173 | 0.02% | 0.00% | 0.00% | 0.00% | 0.08% | 0.03% | 0.01% | 0.01% |
| 174 | 0.02% | 0.00% | 0.00% | 0.00% | 0.08% | 0.03% | 0.01% | 0.01% |
| 175 | 0.02% | 0.00% | 0.00% | 0.00% | 0.08% | 0.03% | 0.01% | 0.01% |
| 176 | 0.02% | 0.00% | 0.00% | 0.00% | 0.08% | 0.03% | 0.01% | 0.01% |
| 177 | 0.02% | 0.00% | 0.00% | 0.00% | 0.08% | 0.03% | 0.01% | 0.01% |
| 178 | 0.02% | 0.00% | 0.00% | 0.00% | 0.08% | 0.03% | 0.01% | 0.01% |
| 179 | 0.02% | 0.00% | 0.00% | 0.00% | 0.07% | 0.03% | 0.01% | 0.01% |
| 180 | 0.02% | 0.00% | 0.00% | 0.00% | 0.07% | 0.03% | 0.01% | 0.01% |
| 181 | 0.02% | 0.00% | 0.00% | 0.00% | 0.07% | 0.03% | 0.01% | 0.01% |
| 182 | 0.02% | 0.00% | 0.00% | 0.00% | 0.07% | 0.03% | 0.01% | 0.01% |
| 183 | 0.02% | 0.00% | 0.00% | 0.00% | 0.07% | 0.03% | 0.01% | 0.01% |
| 184 | 0.02% | 0.00% | 0.00% | 0.00% | 0.07% | 0.02% | 0.01% | 0.01% |
| 185 | 0.02% | 0.00% | 0.00% | 0.00% | 0.06% | 0.02% | 0.01% | 0.01% |
| 186 | 0.02% | 0.00% | 0.00% | 0.00% | 0.06% | 0.02% | 0.01% | 0.01% |
| 187 | 0.01% | 0.00% | 0.00% | 0.00% | 0.06% | 0.02% | 0.01% | 0.01% |
| 188 | 0.01% | 0.00% | 0.00% | 0.00% | 0.06% | 0.02% | 0.01% | 0.01% |
| 189 | 0.01% | 0.00% | 0.00% | 0.00% | 0.06% | 0.02% | 0.00% | 0.01% |
| 190 | 0.01% | 0.00% | 0.00% | 0.00% | 0.06% | 0.02% | 0.00% | 0.01% |
| 191 | 0.01% | 0.00% | 0.00% | 0.00% | 0.06% | 0.02% | 0.00% | 0.01% |
| 192 | 0.01% | 0.00% | 0.00% | 0.00% | 0.06% | 0.02% | 0.00% | 0.01% |
| 193 | 0.01% | 0.00% | 0.00% | 0.00% | 0.05% | 0.02% | 0.00% | 0.01% |
| 194 | 0.01% | 0.00% | 0.00% | 0.00% | 0.05% | 0.02% | 0.00% | 0.01% |
| 195 | 0.01% | 0.00% | 0.00% | 0.00% | 0.05% | 0.02% | 0.00% | 0.01% |
| 196 | 0.01% | 0.00% | 0.00% | 0.00% | 0.05% | 0.02% | 0.00% | 0.01% |
| 197 | 0.01% | 0.00% | 0.00% | 0.00% | 0.05% | 0.02% | 0.00% | 0.00% |
| 198 | 0.01% | 0.00% | 0.00% | 0.00% | 0.05% | 0.02% | 0.00% | 0.00% |
| 199 | 0.01% | 0.00% | 0.00% | 0.00% | 0.05% | 0.02% | 0.00% | 0.00% |
| 200 | 0.01% | 0.00% | 0.00% | 0.00% | 0.05% | 0.02% | 0.00% | 0.00% |

| **Supplemental document: Table S3. Scenario analysis (USD*)** | |  |  |  |
| --- | --- | --- | --- | --- |
| **No** | **Scenario** | **Range** | **Cost/QALY:S-1 vs FFX** | |
|  |  |  | **Lower or variable** | **Upper** |
| 0 | Basic analysis | 0% | 84,451 | - |
| 1 | Monthly medical costs for S-1 in SD | ±10% | 86,056 | 82,847 |
| 2 | Monthly medical costs for FFX in SD | ±10% | 75,490 | 93,413 |
| 3 | Monthly medical costs for S-1 in PD | ±10% | 86,916 | 81,986 |
| 4 | Monthly medical costs for FFX in PD | ±10% | 80,898 | 88,004 |
| 5 | QoL score for S-1 in SD | ±10% | 78,943 | 90,786 |
| 6 | QoL score for FFX in SD | ±10% | 101,284 | 72,416 |
| 7 | QoL score for S-1 in PD | ±10% | 82,952 | 86,006 |
| 8 | QoL score for FFX in PD | ±10% | 86,057 | 82,905 |
| 9 | Varying analysis period | 12 months | 94,674 | - |
| 10 | Varying discount rate | 3% | 84,477 | - |
| 11 | Varying frequency of adverse event | 10 times | 96,931 | - |
| 12 | QOL value for PD if QOL value for general population | -0.119 | 85,342 | - |
| *On 10 July 2025, the exchange rate of 1 USD to yen was 146.26 | |  |  |  |

| **Supplemental document: Figure S1. Comparison of treatment regimens other than S-1 (USD*)** | | | | | | | |
| --- | --- | --- | --- | --- | --- | --- | --- |
| A) Cost/QALY |  |  |  |  |  |  |  |
| **FFX*** |  |  |  |  |  |  |  |
| 39,074 | **GnP** |  |  |  |  |  |  |
| 74,868 | 121,723 | **GEM** |  |  |  |  |  |
|  |  |  |  |  |  |  |  |
| B) Cost/LY |  |  |  |  |  |  |  |
| **FFX*** |  |  |  |  |  |  |  |
| 24,409 | **GnP** |  |  |  |  |  |  |
| 40,369 | 55,662 | **GEM** |  |  |  |  |  |
|  |  |  |  |  |  |  |  |
| *On 10 July 2025, the exchange rate of 1 USD to yen was 146.26 | | | |  |  |  |  |
